# Supplementary material for: Diagnostic Evaluation of an Increased Risk of Developing Small Intestinal Bacterial Overgrowth Associated with Glucagon-like Peptide-1 (GLP-1) Receptor Agonists and Dual GLP-1/GIP Receptor Agonists: A Global Retrospective Multicenter Cohort Analysis
Source: Diagnostics (Basel). 2025 Sep 7;15(17):2264. doi: 10.3390/diagnostics15172264 (PMC12427755; doi:10.3390/diagnostics15172264)
Supplement: Supplementary file 1 [file diagnostics-15-02264-s001.zip › diagnostics-3796857-supplementary.pdf]

diagnostics

MDPI

Supplementary Table S1. Expanded Baseline Characteristics

Continuous variables summarized as mean (SD) or median (IQR) if skewed; pre-match comparisons use Welch's t-test (or Wilcoxon). Categorical variables summarized as n (%); pre-match comparisons use Pearson's χ^2 (or Fisher's exact when expected cell counts <5). Post-match covariate balance is assessed by absolute standardized mean differences (SMD); SMD < 0.10 indicates acceptable balance. Cells with ≤10 are suppressed per data-use policy.

S1a. Continuous variables

Variable	Pre GLP-1 Mean±SD	Pre Control Mean±SD	Welch t	df	p-value	Post GLP-1 Mean±SD	Post Control Mean±SD	SMD (absolute, post)
Age, years	55.6 ± 13.50	62.5 ± 12.60	-224.67	342391	0.0e+00	56.7 ± 13.00	56.7 ± 13.40	0.000
BMI, kg/m²	37.2 ± 8.33	31.5 ± 7.52	302.51	335106	0.0e+00	36.4 ± 7.90	35.9 ± 8.31	0.062
HbA1c, %	7.8 ± 2.16	8.1 ± 2.18	-62.16	361696	0.0e+00	7.9 ± 2.17	8.0 ± 2.25	0.032

S1b. Categorical variables

Variable	Pre n/N (GLP-1)	Pre % (GLP-1)	Pre n/N (Control)	Pre % (Control)	χ^2	p-value	SMD (absolute, pre)	Post n/N (GLP-1)	Post % (GLP-1)	Post n/N (Control)	Post % (Control)	SMD (absolute, post)
Female	130070/234701	55.4%	394335/952418	41.4%	14999.78	0.0e+00	0.283	115650/216173	53.5%	115379/216173	53.4%	0.003
Male	94047/234701	40.1%	535217/952418	56.2%	19654.47	0.0e+00	0.327	91358/216173	42.3%	91454/216173	42.3%	0.001
Hispanic/LatinX	19273/234701	8.2%	86609/952418	9.1%	180.26	4.3e-41	0.031	18213/216173	8.4%	17304/216173	8.0%	0.015
Not Hispanic/LatinX	137998/234701	58.8%	485443/952418	51.0%	4626.93	0.0e+00	0.158	125595/216173	58.1%	131526/216173	60.8%	0.056
Unknown/Not recorded (ethnicity)	77430/234701	33.0%	380366/952418	39.9%	3834.51	0.0e+00	0.145	72365/216173	33.5%	67343/216173	31.1%	0.050
Asian	8366/234701	3.6%	79874/952418	8.4%	6362.96	0.0e+00	0.204	8231/216173	3.8%	7947/216173	3.7%	0.007
Black	44108/234701	18.8%	132399/952418	13.9%	3559.99	0.0e+00	0.133	39336/216173	18.2%	39746/216173	18.4%	0.005
White	129966/234701	55.4%	459037/952418	48.2%	3881.11	0.0e+00	0.144	119423/216173	55.2%	123649/216173	57.2%	0.039
American Indian/Alaska Native	792/234701	0.3%	2606/952418	0.3%	26.88	2.2e-07	0.012	741/216173	0.3%	577/216173	0.3%	0.014
Native Hawaiian/Other	1430/234701	0.6%	5888/952418	0.6%	0.25	0.621	0.001	1332/216173	0.6%	1566/216173	0.7%	0.013

Pacific Islander												
Unknown/Not recorded (race)	40556/234701	17.3%	234549/952418	24.6%	5708.69	0.0e+00	0.181	38337/216173	17.7%	34059/216173	15.8%	0.053
Primary hypertension	159587/234701	68.0%	640293/952418	67.2%	50.49	1.2e-12	0.016	148292/216173	68.6%	148742/216173	68.8%	0.004
Hyperlipidemia	109315/234701	46.6%	401625/952418	42.2%	1491.94	0.0e+00	0.089	100947/216173	46.7%	101353/216173	46.9%	0.004
ASCVD (native coronary artery)	31441/234701	13.4%	205487/952418	21.6%	7885.35	0.0e+00	0.217	30824/216173	14.3%	30893/216173	14.3%	0.001
Other hypothyroidism	35475/234701	15.1%	91180/952418	9.6%	6066.88	0.0e+00	0.169	30643/216173	14.2%	30511/216173	14.1%	0.002
T2DM with neurological complications	28515/234701	12.2%	95272/952418	10.0%	928.72	5.6e-204	0.068	26598/216173	12.3%	26714/216173	12.4%	0.002
Chronic kidney disease	26023/234701	11.1%	147717/952418	15.5%	2947.10	0.0e+00	0.131	25181/216173	11.7%	25195/216173	11.7%	0.000
Heart failure	16791/234701	7.2%	129371/952418	13.6%	7209.18	0.0e+00	0.212	16593/216173	7.7%	17064/216173	7.9%	0.008
Other functional intestinal disorders	18922/234701	8.1%	57691/952418	6.1%	1253.66	1.3e-274	0.078	16232/216173	7.5%	15598/216173	7.2%	0.011
Peripheral vascular disease, unspecified	8504/234701	3.6%	42087/952418	4.4%	292.15	1.7e-65	0.041	8128/216173	3.8%	7986/216173	3.7%	0.003
Atherosclerosis	8079/234701	3.4%	40400/952418	4.2%	307.34	8.3e-69	0.042	7757/216173	3.6%	7631/216173	3.5%	0.003
Cerebral infarction	7801/234701	3.3%	50448/952418	5.3%	1570.98	0.0e+00	0.097	7609/216173	3.5%	7467/216173	3.5%	0.004
End stage renal disease	3604/234701	1.5%	17977/952418	1.9%	130.67	2.9e-30	0.027	3414/216173	1.6%	3417/216173	1.6%	0.000
Fibrosis/cirrhosis of liver	2979/234701	1.3%	13675/952418	1.4%	37.76	8.0e-10	0.014	2824/216173	1.3%	2667/216173	1.2%	0.006
Other immunodeficiencies	2369/234701	1.0%	4166/952418	0.4%	1125.18	1.1e-246	0.068	1839/216173	0.8%	1984/216173	0.9%	0.007
Malnutrition	1716/234701	0.7%	14370/952418	1.5%	851.89	2.8e-187	0.074	1671/216173	0.8%	1505/216173	0.7%	0.009

Opioid dependence	1762/234701	0.8%	4262/952418	0.5%	342.98	1.4e-76	0.039	1515/216173	0.7%	1500/216173	0.7%	0.001
HIV disease	1376/234701	0.6%	3418/952418	0.4%	242.10	1.4e-54	0.033	1189/216173	0.6%	1271/216173	0.6%	0.005
Drug-induced constipation	1078/234701	0.5%	2148/952418	0.2%	379.72	1.4e-84	0.040	894/216173	0.4%	875/216173	0.4%	0.001
Celiac disease	614/234701	0.3%	658/952418	0.1%	652.05	8.0e-144	0.047	442/216173	0.2%	289/216173	0.1%	0.017
Other chronic pancreatitis	408/234701	0.2%	3720/952418	0.4%	255.28	1.8e-57	0.041	404/216173	0.2%	467/216173	0.2%	0.006
Ab antibody defects (immunodeficiency)	361/234701	0.1%	812/952418	0.1%	89.65	2.8e-21	0.020	305/216173	0.1%	249/216173	0.1%	0.007
Diverticular disease of small intestine	183/234701	0.1%	776/952418	0.1%	0.29	0.592	0.001	172/216173	0.1%	154/216173	0.1%	0.003
Common variable immunodeficiency	100/234701	0.0%	181/952418	0.0%	nan	nan	0.013	83/216173	0.0%	65/216173	0.0%	0.005
Acromegaly/pituitary gigantism	92/234701	0.0%	194/952418	0.0%	nan	nan	0.011	76/216173	0.0%	67/216173	0.0%	0.002
Alcohol-induced chronic pancreatitis	42/234701	0.0%	155/952418	0.0%	nan	nan	0.001	42/216173	0.0%	92/216173	0.0%	0.013
Achlorhydria	39/234701	0.0%	47/952418	0.0%	nan	nan	0.011	36/216173	0.0%	17/216173	0.0%	0.008
Myotonic muscular dystrophy	25/234701	0.0%	111/952418	0.0%	nan	nan	0.001	23/216173	0.0%	28/216173	0.0%	0.002
Combined immunodeficiencies	21/234701	0.0%	58/952418	0.0%	nan	nan	0.003	16/216173	0.0%	15/216173	0.0%	0.001
Tropical sprue	10/234701	0.0%	12/952418	0.0%	nan	nan	0.006	10/216173	0.0%	10/216173	0.0%	0.000
Gastroenteritis/colitis due to radiation	11/234701	0.0%	46/952418	0.0%	nan	nan	0.000	10/216173	0.0%	12/216173	0.0%	0.001

Systemic sclerosis (scleroderma)	0/234701	0.0%	0/952418	0.0%	nan	nan	nan	0/216173	0.0%	0/216173	0.0%	nan
Amyloidosis	0/234701	0.0%	0/952418	0.0%	nan	nan	nan	0/216173	0.0%	0/216173	0.0%	nan
Gastroparesis	0/234701	0.0%	0/952418	0.0%	nan	nan	nan	0/216173	0.0%	0/216173	0.0%	nan
BMI ⩾ 25 kg/m²	168304/234701	71.7%	492218/952418	51.7%	30605.06	0.0e+00	0.421	150141/216173	69.5%	150743/216173	69.7%	0.006
BMI ⩾ 30 kg/m²	150585/234701	64.2%	345445/952418	36.3%	60213.41	0.0e+00	0.581	132446/216173	61.3%	132557/216173	61.3%	0.001
BMI ⩾ 35 kg/m²	111793/234701	47.6%	188694/952418	19.8%	77087.11	0.0e+00	0.616	94275/216173	43.6%	94681/216173	43.8%	0.004
BMI ⩾ 40 kg/m²	70047/234701	29.9%	95351/952418	10.0%	61770.84	0.0e+00	0.513	55850/216173	25.8%	56129/216173	26.0%	0.003
BMI ⩾ 45 kg/m²	38925/234701	16.6%	44772/952418	4.7%	40580.23	0.0e+00	0.393	29328/216173	13.6%	29483/216173	13.6%	0.002
BMI ⩽ 50 kg/m²	164607/234701	70.1%	554881/952418	58.3%	11120.97	0.0e+00	0.250	149230/216173	69.0%	148578/216173	68.7%	0.007
BMI ⩽ 45 kg/m²	155179/234701	66.1%	542021/952418	56.9%	6586.61	0.0e+00	0.190	141532/216173	65.5%	140779/216173	65.1%	0.007
BMI ⩽ 40 kg/m²	135445/234701	57.7%	508701/952418	53.4%	1401.59	9.5e-307	0.087	124545/216173	57.6%	123635/216173	57.2%	0.009
BMI ⩽ 35 kg/m²	100515/234701	42.8%	439347/952418	46.1%	828.41	3.6e-182	0.066	93690/216173	43.3%	92811/216173	42.9%	0.008
BMI ⩽ 30 kg/m²	52960/234701	22.6%	298935/952418	31.4%	7026.87	0.0e+00	0.200	50079/216173	23.2%	49462/216173	22.9%	0.007
HbA1c ⩾ 7.0%	105322/234701	44.9%	357521/952418	37.5%	4260.92	0.0e+00	0.149	98200/216173	45.4%	96673/216173	44.7%	0.014
HbA1c ⩾ 7.5%	87847/234701	37.4%	298785/952418	31.4%	3146.74	0.0e+00	0.128	82081/216173	38.0%	81397/216173	37.6%	0.007
HbA1c ⩾ 8.0%	75221/234701	32.0%	248228/952418	26.1%	3404.64	0.0e+00	0.132	70059/216173	32.4%	69792/216173	32.3%	0.003
HbA1c ⩾ 8.5%	64362/234701	27.4%	203207/952418	21.3%	3996.16	0.0e+00	0.142	59668/216173	27.6%	59585/216173	27.6%	0.001
HbA1c ⩾ 9.0%	56002/234701	23.9%	170295/952418	17.9%	4365.38	0.0e+00	0.148	51692/216173	23.9%	51718/216173	23.9%	0.000
HbA1c ⩾ 9.5%	47943/234701	20.4%	141157/952418	14.8%	4419.45	0.0e+00	0.148	44070/216173	20.4%	44053/216173	20.4%	0.000
HbA1c ⩾ 10.0%	41338/234701	17.6%	118895/952418	12.5%	4243.48	0.0e+00	0.144	37856/216173	17.5%	37817/216173	17.5%	0.000
HbA1c ⩽ 11.0%	150745/234701	64.2%	432191/952418	45.4%	26772.02	0.0e+00	0.386	133601/216173	61.8%	129609/216173	60.0%	0.038

HbA1c ⩽ 10.5%	148443/234701	63.2%	422823/952418	44.4%	26809.16	0.0e+00	0.385	131369/216173	60.8%	127446/216173	59.0%	0.037
HbA1c ⩽ 10.0%	145620/234701	62.0%	411384/952418	43.2%	26868.25	0.0e+00	0.384	128625/216173	59.5%	124702/216173	57.7%	0.037
HbA1c ⩽ 9.5%	142545/234701	60.7%	399096/952418	41.9%	26915.54	0.0e+00	0.384	125658/216173	58.1%	121680/216173	56.3%	0.037
HbA1c ⩽ 9.0%	138532/234701	59.0%	383069/952418	40.2%	27030.16	0.0e+00	0.383	121816/216173	56.4%	117808/216173	54.5%	0.037
HbA1c ⩽ 8.5%	133853/234701	57.0%	363584/952418	38.2%	27502.35	0.0e+00	0.384	117348/216173	54.3%	113434/216173	52.5%	0.036
HbA1c ⩽ 8.0%	129033/234701	55.0%	343442/952418	36.1%	28125.51	0.0e+00	0.387	112735/216173	52.1%	108934/216173	50.4%	0.035
Opioid analgesics	108932/234701	46.4%	381176/952418	40.0%	3172.97	0.0e+00	0.129	98570/216173	45.6%	99173/216173	45.9%	0.006
Proton pump inhibitors	71525/234701	30.5%	267157/952418	28.1%	542.86	4.5e-120	0.053	64820/216173	30.0%	65383/216173	30.2%	0.006
Dicyclomine	4813/234701	2.0%	9964/952418	1.1%	1545.64	0.0e+00	0.081	3952/216173	1.8%	3864/216173	1.8%	0.003
Loperamide	3068/234701	1.3%	11347/952418	1.2%	21.05	4.5e-06	0.010	2749/216173	1.3%	2600/216173	1.2%	0.006
Diphenoxylate	1123/234701	0.5%	2909/952418	0.3%	166.58	4.1e-38	0.028	945/216173	0.4%	940/216173	0.4%	0.000

Supplementary Table S2: STROBE Statement—Checklist for Cohort Studies

**Section/Topic**	**Item No.**	**STROBE Recommendation**	**Where addressed in manuscript**	**Notes**
Title/Abstract	1a	Indicate the study's design with a commonly used term in the title or the abstract.	Title page; Structured Abstract	
Title/Abstract	1b	Provide in the abstract an informative and balanced summary of what was done and what was found.	Structured Abstract	
Introduction	2	Explain the scientific background and rationale for the investigation.	Introduction §Background	
Introduction	3	State specific objectives and any prespecified hypotheses.	Introduction §Objectives	
Methods	4	Present key elements of study design early in the paper.	Methods §2.1 Data Source; Methods §2.2 Patient Selection; Methods §2.5 Study Outcomes	
Methods	5	Describe the setting, locations, and relevant dates, including periods of recruitment, exposure, follow-up, and data collection.	Methods §2.1 Data Source; Methods §2.2 Patient Selection (index from Jan 1, 2006; follow-up horizons 3, 6, 12, 60 months)	
Methods	6a	Give the eligibility criteria, and the sources and methods of selection of participants.	Methods §2.2 Patient Selection	
Methods	6b	For matched studies, give matching criteria and number of exposed and unexposed.	Methods §2.4 Covariates and Matching (1:1 greedy nearest neighbor; caliper 0.10; matched n=216,173 per cohort)	
Methods	7	Clearly define all outcomes, exposures, predictors, potential confounders, and effect modifiers. Give diagnostic criteria, if applicable.	Methods §2.5 Study Outcomes; Methods §2.4 Covariates and Matching (covariates listed); Methods §2.3 Query Diagnostic Validation (breath test & treatment confirmation)	
Methods	8*	For each variable of interest, give sources of data and details of assessment (measurement).	Methods §2.1 Data Source; coding per Table 1; Methods §2.3 Query Diagnostic Validation	

Methods	9	Describe any efforts to address potential sources of bias.	Methods §2.4 Covariates and Matching (PSM, SMD<0.10); Methods §2.6 Statistical Analysis (exact tests for zero/small cells); Methods §2.1 Data Source (small-cell suppression)	
Methods	10	Explain how the study size was arrived at.	All eligible adults within TriNetX during study window; matched sample sizes reported in Results/Flow Diagram.	
Methods	11	Explain how quantitative variables were handled in the analyses. If applicable, describe which groupings were chosen and why.	Methods § 2.6 Statistical Analysis (means/SD, thresholds e.g., BMI ⩾ 30); Table 2; Supplementary Table S1	
Methods	12a	Describe all statistical methods, including those used to control for confounding.	Methods §2.6 Statistical Analysis (PSM caliper 0.10; Welch's t, χ^2/Fisher; Poisson IRs/IRR; KM/log-rank; univariate Cox; exact tests for zero events)	
Methods	12b	Describe any methods used to examine subgroups and interactions.	Methods §2.6 Statistical Analysis (subcohorts at 3/6/12 months; long-term 60 months; persistence subset)	
Methods	12c	Explain how missing data were addressed.	TriNetX provides aggregate counts; small-cell suppression (⩽ 10). Missingness discussed in Limitations.	
Methods	12d	If applicable, explain how loss to follow-up was addressed.	Methods §2.5 Study Outcomes (censoring at last encounter/death or horizon end)	
Methods	12e	Describe any sensitivity analyses.	Results/Discussion (persistence subset analyses as sensitivity).	
Results	13a	Report numbers of individuals at each stage of study (e.g., eligibility, matching, follow-up, analysis).	Figure 1 (Cohort flow diagram)	
Results	13b	Give reasons for non-participation at each stage.	Figure 1 (Cohort flow diagram) (as applicable within TriNetX aggregate data)	
Results	13c	Consider use of a flow diagram.	Figure 1 (Cohort flow diagram)	

Results	14a	Give characteristics of study participants (e.g., demographic, clinical, social) and information on exposures and potential confounders.	Table 2; Supplementary Table S1	
Results	14b	Indicate number of participants with missing data for each variable of interest.	Reported implicitly via available counts; small-cell suppression annotated in tables.	
Results	15	Report numbers of outcome events or summary measures over time.	Results §Primary and Secondary Analyses (incidence rates per 1,000 person-years; KM; Cox HRs)	
Results	16a	Give unadjusted estimates and, if applicable, confounder-adjusted estimates and their precision (e.g., 95% CI).	Results §Primary and Secondary Analyses (matched estimates with 95% CI); Methods §2.6 Statistical Analysis	
Results	16b	Report category boundaries when continuous variables were categorized.	Table 2; Supplementary Table S1 (e.g., BMI $\geqslant$ 30 kg/m^2, HbA1c thresholds)	
Results	16c	If relevant, consider translating estimates of relative risk into absolute risk for a meaningful time period.	Incidence rates presented per 1,000 person-years in Results.	
Results	17	Report other analyses done—e.g., analyses of subgroups and sensitivity analyses.	Persistence/landmark-style subsets at 3/6/12 months; 3–60-month window.	
Discussion	18	Summarize key results with reference to study objectives.	Discussion—Key Results	
Discussion	19	Discuss limitations of the study, taking into account sources of potential bias or imprecision.	Discussion—Limitations, data-source constraints, zero/small cells, unmeasured confounding	
Discussion	20	Give a cautious overall interpretation of results considering objectives, limitations, multiplicity of analyses, results from similar studies, and other relevant evidence.	Discussion—Interpretation	
Discussion	21	Discuss the generalizability (external validity) of the study results.	Discussion—Generalisability	

Other information	22	Give the source of funding and the role of the funders for the present study.	Funding/Support (add if applicable)	

Abbreviations: KM, Kaplan–Meier; IR, incidence rate; IRR, incidence rate ratio; PSM, propensity score matching; SMD, standardized mean difference; TriNetX small-cell suppression policy applied (≤10).
